# Analysing the role of complexity in explaining the fortunes of technology programmes: empirical application of the NASSS framework

**DOI:** 10.1186/s12916-018-1050-6

**Published:** 2018-05-14

**Authors:** Trisha Greenhalgh, Joe Wherton, Chrysanthi Papoutsi, Jenni Lynch, Gemma Hughes, Christine A’Court, Sue Hinder, Rob Procter, Sara Shaw

**Affiliations:** 1 Department of Primary Care Health Sciences, University of Oxford, Oxford, OX2 6GG UK; 2 School of Health and Social Work, University of Hertfordshire, Hatfield, UK; 3 RAFT Research consultancy, Clitheroe, UK; 4 Department of Computer Science, University of Warwick, Coventry, UK

## Abstract

**Background:**

Failures and partial successes are common in technology-supported innovation programmes in health and social care. Complexity theory can help explain why. Phenomena may be simple (straightforward, predictable, few components), complicated (multiple interacting components or issues) or complex (dynamic, unpredictable, not easily disaggregated into constituent components). The recently published NASSS framework applies this taxonomy to explain *N*on-adoption or *A*bandonment of technology by individuals and difficulties achieving *S*cale-up, *S*pread and *S*ustainability. This paper reports the first empirical application of the NASSS framework.

**Methods:**

Six technology-supported programmes were studied using ethnography and action research for up to 3 years across 20 health and care organisations and 10 national-level bodies. They comprised video outpatient consultations, GPS tracking technology for cognitive impairment, pendant alarm services, remote biomarker monitoring for heart failure, care organising software and integrated case management via data warehousing. Data were collected at three levels: micro (individual technology users), meso (organisational processes and systems) and macro (national policy and wider context). Data analysis and synthesis were guided by socio-technical theories and organised around the seven NASSS domains: (1) the condition or illness, (2) the technology, (3) the value proposition, (4) the adopter system (professional staff, patients and lay carers), (5) the organisation(s), (6) the wider (institutional and societal) system and (7) interaction and mutual adaptation among all these domains over time.

**Results:**

The study generated more than 400 h of ethnographic observation, 165 semi-structured interviews and 200 documents. The six case studies raised multiple challenges across all seven domains. Complexity was a common feature of all programmes. In particular, individuals’ health and care needs were often complex and hence unpredictable and ‘off algorithm’. Programmes in which multiple domains were *complicated* proved difficult, slow and expensive to implement. Those in which multiple domains were *complex* did not become mainstreamed (or, if mainstreamed, did not deliver key intended outputs).

**Conclusion:**

The NASSS framework helped explain the successes, failures and changing fortunes of this diverse sample of technology-supported programmes. Since failure is often linked to complexity across multiple NASSS domains, further research should systematically address ways to reduce complexity and/or manage programme implementation to take account of it.

## Background

### Introduction

Technological innovation is viewed by policymakers as a driver of both health and wealth [1]. Technology is often depicted as ’empowering’ for both patients and staff, and has been associated with improved efficiency, quality and safety of care [2–5]. In reality, however, technology start-ups may fail to attract investment [6]; patients may or may not be able or willing to use new technologies [7]; professionals may resist them [8–10]; new technologies may clash with legacy systems and with established routines [11, 12]; a technology may be implemented but fail to deliver the anticipated benefits [13]; and small-scale demonstration projects may fail to scale up locally, spread distantly or be sustained over time [14, 15].

In a recently published systematic review, we synthesised evidence on individual, team, organisational and system influences on the success of technology-supported innovation programmes in health and social care [16]. We drew in particular on published technology implementation frameworks and key theoretical work on diffusion of innovations [17, 18], technological entrepreneurship [6, 19], the patient experience of chronic illness [20], clinician resistance to technologies [21], the social processes of ‘normalising’ technologies in organisations [22–25], business and financial planning [14], organisational resilience and sustainability [26–28], and theoretical studies on complex adaptive systems [29, 30].

**Fig. 1:**
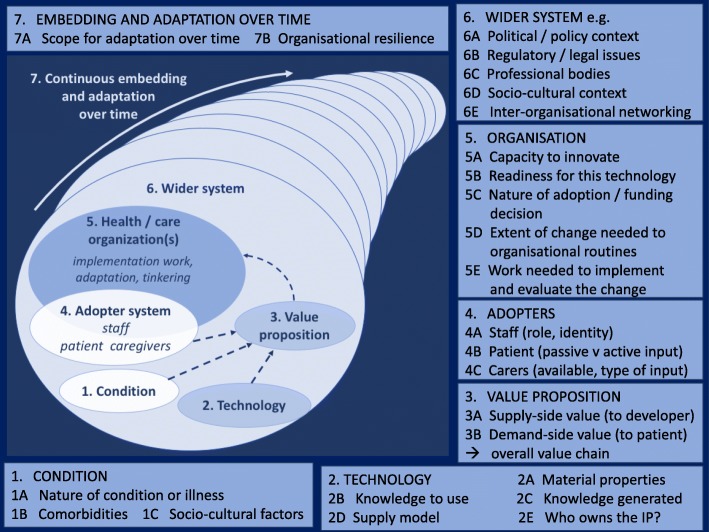
The NASSS framework for considering influences on the adoption, non-adoption, abandonment, spread, scale-up and sustainability of health and care technologies. Image adapted from J Med Internet Res. 2017; 19: e367

Our synthesis of this diverse literature occurred in parallel with testing of candidate domains and theories from our systematic review on a sample of six empirical case studies. We produced a new multi-level interdisciplinary framework called NASSS (*N*on-adoption or *A*bandonment of technology by individuals and difficulties achieving *S*cale-up, *S*pread and *S*ustainability), which incorporates and extends the theoretical frameworks and models listed in the previous paragraph. The NASSS framework is shown diagrammatically in Panel 1 and Fig. 1.

**Table:** 

Panel 1: Domains and questions in the NASSS framework Domain 1: the condition 1A. What is the nature of the condition or illness? 1B. What are the relevant co-morbidities? 1C. What are the relevant socio-cultural factors? Domain 2: the technology 2A. What are the key features of the technology? 2B. What kind of knowledge does the technology bring into play? 2C. What knowledge and/or support is required to use the technology? 2D. What is the technology supply model? 2E. Who owns the intellectual property (IP) generated by the technology? Domain 3: the value proposition and value chain 3A. What is the developer’s business case for the technology (supply-side value)? 3B. What is its desirability, efficacy, safety and cost-effectiveness (demand-side value)? Domain 4: the adopter system 4A. What changes in staff roles, practices and identities are implied? 4B. What is expected of the patient (and/or immediate carer) — and is this achievable by and acceptable to them? 4C. What is assumed about the extended network of lay carers? Domain 5: the organisation(s) 5A. What is the organisation’s capacity to innovate? 5B. How ready is the organisation for this technology-supported change? 5C. How easy will the adoption and funding decision be? 5D. What changes will be needed in team interactions and routines? 5E. What work is involved in implementation and who will do it? Domain 6: the wider system 6A. What is the political context for programme development, implementation and roll-out? 6B. What is the regulatory context? 6C. What is the position of professional bodies? 6D. What is the socio-cultural context (public perception, interest, expectation)? 6E. What is the nature and extent of inter-organisational networking? Domain 7: Embedding and adaptation over time 7A. How much scope is there for adapting and co-evolving the technology and the service over time? 7B. How resilient is the organisation to handling critical events and adapting to unforeseen eventualities?

The original questions guiding our empirical research were the following. (1) How can we improve the process by which health and care technologies are developed and implemented? (2) How can we support the customisation and use of such technologies in the home and/or the health or care setting? (3) How can we ensure that patients’ needs and concerns remain central when developing technology-supported service innovations [31, 32]? The study of complexity was not part of our original proposal, but it quickly emerged as the dominant theme in our empirical data (as well as a prominent element in the more recent literature we were identifying for our systematic review [29, 30]).

Our previous publication focused mainly on the secondary research component of the NASSS framework [16]. This paper presents a more detailed account of our empirical findings and illustrates how the framework allowed us to explore complexity in multiple interacting domains.

### Complex systems and the NASSS framework

A system is defined as an assembly of agents that interact with each other. In a simple system (few agents and components) or a complicated one (many agents and components), the relationships between agents are well defined and stable, which means the overall behaviour of the system is predictable. In contrast, complex, adaptive systems are composed of agents with ill-defined and unstable boundaries that may act in unexpected ways, whose actions are interconnected so that one agent’s actions change the context for other agents [33]. Hence, in *complex* systems, agents interact with each other and with other systems in unexpected ways; their component agents (people, cells, technologies) can simultaneously be members of several systems. The complex system works by a fundamentally different logic, since its response to change is—to a greater or lesser extent—unpredictable and non-linear [34].

Against this background, the NASSS framework has been developed to encourage complex thinking (what Tsoukas calls ‘conjunctive theorising’ [35]) about technological innovations in healthcare. With a view to generating a rich narrative of events unfolding in a real-world setting, aspects of the different domains are first considered in terms of whether they are simple (straightforward, predictable, few components—as in making a sandwich), complicated (remains predictable but there are multiple interacting components or issues—as in building a rocket) or complex (dynamic, unpredictable, not easily disaggregated into constituent components—as in raising a child). Simple and complicated phenomena operate according to linear, Newtonian (predictive, cause-and-effect) logic; they can, for example, be meaningfully analysed in terms of their component parts. Complex phenomena operate according to different (non-linear) logic, in which a given cause may not always have the same effect. They exhibit broad patterns and emerge over time, but they are not predictable.

A simple illness or condition (domain 1 in the NASSS framework) is well characterised, well understood and predictable (though it may still be *serious*, e.g. deep venous thrombosis); its management is straightforward and is influenced only minimally by co-morbidities or socio-cultural factors. The same goes for a complicated condition (e.g. many cancers), though the logistics may be more challenging. Complex conditions, in contrast, are poorly characterised, poorly understood, unpredictable and/or strongly influenced by co-morbidities and socio-cultural factors (e.g. drug dependency, dementia).

The complexity dimension in domain 2 (technology) may refer to the technology’s material (including technical) properties, its ease of use, the kind of data it generates, its supply chain or the intellectual property associated with it. In all these sub-domains, complexity—which is impossible to define in rigid and universal terms—may relate to changeability, unpredictability, contestability (e.g. experts disagree on what the data mean and whether they can be trusted) and interdependence with other changeable, unpredictable or contestable aspects (such as availability of broadband).

The value proposition (domain 3) refers to both supply-side value (whether there is a straightforward and uncontested business case for generating revenue for the developer) and demand-side value (whether there is strong and uncontested evidence that the technology is desirable for patients, effective, safe and cost-effective). Complexity in this domain relates to (for example) multiple and perhaps interdependent assumptions on which the business case is based, a speculative or contested evidence base for effectiveness or cost-effectiveness, or gaps in the overall value chain.

Complexity in relation to intended adopters (domain 4) does not mean merely that some individuals need to learn new skills or procedures or adopt new staff roles. More challenging is the expectation that a staff member, patient or lay carer will need to take on a different identity (e.g. data processer, teacher) alongside their traditional one and/or make judgements that are difficult or unpredictable.

Complexity in the organisational setting (domain 5) relates in particular to the scope, scale, pace, resource requirements, and the logistical uncertainties and interdependencies of delivering the innovation and the associated new service model [36]. The introduction of a ’disruptive’ technology (that is, one that enables—and perhaps requires—organisational work to be done differently [37]) will be complex if known preconditions for innovation are not met (e.g. if there is weak leadership, poor managerial relations or severely stretched resources); if the technology is a poor strategic ‘fit’; if new work routines and/or inter-organisational co-operation are needed; or if a large amount of work is needed to build a vision, engage staff, implement the programme and develop ways of monitoring its impact.

Complexity in the external context (domain 6) means that there are tricky hurdles to be overcome in relation to political, financial, legal, regulatory or public concerns, or that inter-organisational networking and knowledge sharing are difficult. Again, the key issue is often the interdependency of different influences (which tends to mean that any one problem cannot be addressed without generating other problems elsewhere in the system).

Finally, complexity in domain 7 (adaptation over time) means that further adaptation or co-evolution of the technology is impossible because of lack of material or technical flexibility, and/or because the organisation(s) lack the resilience to adapt to changing external conditions (see Discussion).

Such were the findings of our systematic review that formed the theoretical basis of the NASSS framework [16]. In the remainder of this paper, we describe the empirical testing and refining of the NASSS domains across a maximum-variety sample of technology implementation case studies. The specific research question addressed in this paper was: Given that technology programmes in health and social care are often described as ‘complex’, what is the nature of this complexity and how might it affect the fortunes of a programme?

## Methods

### Context, governance and methodology

The research took place in various field sites across the UK. It embraced two research programmes: VOCAL (Virtual Online Consultations—Advantages and Limitations) and SCALS (Studies in Co-Creating Assisted Living Solutions). VOCAL (funded from 2015 to 2017, with an earlier set-up phase from 2011) was an in-depth study of the development, introduction and local roll-out of remote (video) consultations in three contrasting clinical departments, each on a different geographical site, in a large, multi-site UK hospital trust [38, 39]. SCALS (funded from 2015 to 2020, with some data collected from 2013) is an action research study of the challenges faced by UK health and social care organisations who introduce technology-supported new service models; it includes examples from healthcare (e.g. remote biomarker monitoring, video consultations, technologies for integrating care across organisations) and social care (safety alarms, GPS tracking, care organising apps) [32].

Both VOCAL and SCALS had an external steering group with a lay chair and representation from patients, front-line clinicians, the technology industry and local and national policymakers (including information leads at National Health Service (NHS) England). The VOCAL study also had a separate patient advisory group convened and chaired by a community anthropologist.

Case studies (all of which were drawn from VOCAL and SCALS) were sampled by a combination of responsiveness (health or care organisations sought our input to real-world implementation challenges), convenience (local initiatives caught our interest) and theoretical sampling (later cases were systematically sought to illustrate themes that had come up in our literature review but were not yet represented in our sample). The six prospective case studies reported below have so far been followed for up to 3 years. Additional, theoretically sampled case studies in the SCALS programme (added more recently and not reported here) will be explored in future papers.

Each case study has involved a flexible programme of qualitative interviews and observation (with patients, clinicians, managers, technical designers, commercial partners and—where relevant—investors), analysis of documents (correspondence, business plans, clinical records), ethnography (of technology use by patients/clients and staff, of meetings and events, and of technology design and functionality) and video-recording of both ends of remote consultations [31, 32]. In addition, in order to build up a rich picture of the national context in which technologies evolve, we used a combination of purposive and snowball sampling to identify 45 potential stakeholders from across government (e.g. NHS England), professional organisations (e.g. Royal College of Physicians, Medical Protection Society), patient groups (e.g. National Voices), industry (e.g. Microsoft) and charitable and third sector organisations (e.g. Health Foundation). We invited a maximum variety sample of 39 of these stakeholders to talk informally with the study team, of whom we spoke with 36 (the remaining 3 being uncontactable). We conducted formal semi-structured interviews with a purposive sample of 12 of these stakeholders (ensuring variation of groups and perspectives) and combined this with analysis of approximately 50 key national-level policy and policy-related documents published since 2000.

Data sources for case studies in VOCAL and SCALS used for development and testing of the NASSS framework are summarised in Table 1. The empirical case studies are outlined briefly below and analysed in more detail in the Results section.

**Table 1:** Summary of empirical case studies and data sources (adapted from J Med Internet Res. 2017; 19: e367)

Study site(s)	Technology/ies	Participants	Data sources
Case A. Video outpatient consultations			
A1: Acute hospital trust (3 specialties — diabetes, antenatal, cancer — on different sites) A2: Nurse-led heart failure service run from community hospital	Skype™ (acute hospital) and FaceTime™ (community hospital) together with commercially available blood pressure and heart rate monitors, weighing scales and oximeter	A1: 24 staff (9 clinicians, 10 support staff, 5 managers); 27 patients A2: 10 staff (8 nurses, one manager, one administrator); 8 patients Plus 48 national stakeholders and wider informants on remote consulting	35 formal semi-structured interviews plus ~ 100 informal interviews; 150+ hours of ethnographic observation; 40 videotaped remote consultations (12 diabetes, 6 antenatal diabetes, 12 cancer, 10 heart failure); 500+ emails; 30 local documents, e.g. business plans, protocols; 50 national-level documents
Case B. GPS tracking for cognitive impairment			
Social care organisation in deprived borough in inner London	GPS tracking devices supplied by 5 different technology companies, includes GPS tracking with virtual map and ‘geo-fence’ alert functions	7 index cases; 8 lay carers; 5 formal carers, 3 social care staff; 3 healthcare staff; 3 call centre staff	22 ethnographic visits and ‘go-along’ interviews with index cases (~ 50 h); 15 ethnographic visits with health and social care staff; 6 staff interviews; 5 team meetings; 3 local protocols
Case C. Pendant alarms			
C1: Healthcare commissioning organisation in deprived borough in outer London C2: Social care organisation in mixed borough in the Midlands	In both sites, pendant alarms and base units were supplied by multiple different technology companies and supported by local councils, each with a different set of arrangements with providers and an ‘arms-length management organisation’ alarm support service	C1: 8 index cases; 7 lay carers; 12 professional staff C2: 11 index cases; 9 health/social care staff from frontline service delivery to senior board level; 3 representatives from telecare industry	50 semi-structured and narrative interviews; 61 ethnographic visits (~ 80 h of observation) including needs assessments and reviews; 20 h of observation at team meetings
Case D. Remote biomarker monitoring in heart failure			
Acute hospital trusts in six different cities in UK	Tablet computer and Bluetooth-enabled commercially available sensing devices (blood pressure and heart rate monitor, weighing scales)	7 research staff including principal investigator and research coordinator for SUPPORT-HF trial; 7 clinical staff involved in trial; 4 clinical staff not involved in trial; (to date) 18 patient participants and one spouse	1 patient focus group; 8 patient interviews; 24 additional semi-structured interviews; SUPPORT-HF study protocol and ethics paperwork; material properties and functionality of biomarker database
Case E. Care organising software			
E1: Healthcare commissioning organisation in northern England E2: National carer support charity in UK	Product A: Web-based portal developed by small tech company for use by families to help them organise and coordinate the care of (typically) an older relative Product B: Smartphone app co-designed by carer support charity for same purpose	Product A: 2 technology developers and CEO of technology company; 4 social care commissioners; 30 health and social care staff considering using the device; 4 users of the device, one non-user Product B (to date): 2 members of care charity (including CEO); 10 qualitative case studies of users undertaken by another academic team	22 semi-structured and narrative interviews; 16 h ethnographic observations of meetings; auto-ethnographic testing of functionality and usability of devices; secondary analysis of 3rd party evaluation of Product B
Case F. Data warehouse for integrated case management			
1 acute hospital trust, 1 community health trust, 3 local councils, 3 healthcare commissioning organisations	Integrated data warehouse incorporating predictive risk modelling (in theory interoperable with record systems in participating organisations)	14 staff; 20 patient participants	14 semi-structured interviews; 50 ethnographic visits (~ 80 h); 12 h shadowing community staff; 4 h observation of interdisciplinary meetings; 12 local protocols/documents

### Outline of the six case studies

Case A (video outpatient consultations) included four clinical services in the NHS: adult diabetes, antenatal diabetes and cancer surgery, all using Skype™ [38], and a community-based, nurse-led heart failure service, using predominantly FaceTime™. Video consultation was offered to patients for whom it was judged clinically appropriate by the clinician. There was strong support from senior management and many (though not all) clinicians. Almost all patients volunteering for this option experienced it as convenient, technically straightforward and able to meet their clinical needs. But implementation proved logistically difficult, technically challenging, labour-intensive and slow. Video consulting was considered not clinically appropriate for many patients. By the end of the study period, video consultations had been abandoned in the antenatal diabetes service and put ‘on hold’ in the community heart failure service. In the adult diabetes and cancer surgery services, they continued and were being extended to other clinical services within the trust.

Case B (global positioning system [GPS] tracking for people with cognitive impairment) began when the SCALS team were approached by a local council in a deprived and multi-ethnic inner city borough and asked to help improve the take-up of devices to electronically track people with cognitive impairment who ‘wandered’ outside the home. We worked with the council and with linked voluntary sector groups to implement and adapt a selection of devices and a linked call centre and monitoring service [40, 41]. Whilst several hundred people in the catchment population had cognitive impairment, only 11 were ever identified as eligible for GPS tracking and 7 assented (of which only 3 continue to use the technology). Successful adoption of the technology was found to require a network of extended family and call centre staff who collectively ‘knew’ the client and his or her preferred walking route(s).

Case C (pendant alarms): Pendant alarms are worn around the neck (or on the wrist) and connected to a remote call centre. The client should press the alarm if he/she is in difficulty (e.g. fallen and cannot get up); the call centre will alert either a relative (on a retained contact list) or an emergency service. Pendant alarm services had been in widespread use for some years in two participating organisations in the SCALS study—both urban settings serving a mixed socio-demographic population. Various arrangements were in place for referring clients (including self, GP, social worker and local age charity) and fitting the alarm (typically a commercial supplier). In both sites, alarms were widely supplied and often ‘worked’ as intended, though they depended on a network of carers and professional staff whose collective knowledge of the client allowed them to interpret remote signals (e.g. judge whether a call was an emergency). In many instances, clients did not activate the alarm when care staff and relatives considered that they should have done so.

Case D (telehealth for heart failure) was the qualitative component of a multi-centre randomised controlled trial of biomarker monitoring (weight, blood pressure, heart rate) in heart failure (SUPPORT-HF). All participants in this trial were supplied with a tablet technology through which they could access their biomarker results, trends and educational material [42]. The intervention arm included active communication of results and recommendations to the patient’s general practitioner with the aim of increasing use of recommended medical therapy and improving patients’ well-being; in the control arm, data were available for the general practitioner to access if desired. Across participating sites, clinicians engaged variably with the study, occasionally leading to slower than predicted recruitment. Participants’ use of the technology also varied widely, influenced by various clinical, technical and logistical issues.

Case E (care organising software) followed the fortunes of two software products, each designed to help relatives and friends organise tasks and visits for someone with health and/or care needs. Product A, a web portal, had been developed in-house by a small software company. The business model was to sell the product (at a cost of several thousand pounds) to care organisations that would then provide it to their clients free of charge. Product B was a smartphone app (with linked web portal) that had been developed via publicly funded R&D using co-design methodology by a national carers’ charity; it was made available for individual download (e.g. via the Apple App Store) for £2.99. By the end of the study, very few families were using Product A, but around 7500 were registered to use Product B (a proportion of whom were also receiving a wider package of support from the care charity).

Case F (shared data warehouse for integrated case management of patients at risk of hospital admission) was introduced in 2009 to support a policy of coordinated, multi-disciplinary case management between health and social care services through assessment and care planning. It had been proposed as a solution to the growing challenge of emergency hospital admissions in older people with multiple health and care needs, reflecting national policy [43]. The cross-organisation data warehouse incorporating a predictive risk modelling tool was intended to automate the identification and stratification of people at high risk of hospital admission, and enable shared access to care plans, thus facilitating coordinated action and frequent dialogue between primary and secondary care providers and social services. However, the original vision of ‘integrated care’ achieved *through* the technology was only partially realised because, in practice, high-risk patients were identified and managed through a combination of risk stratification and data entry (using the technology) and clinical judgement and dialogue (bypassing the technology).

### Data analysis and testing of the NASSS framework

We sought to analyse our six case studies both individually [44] and also as a theoretically sampled collection of cases representing maximum diversity in each of the NASSS domains [45]. This work both informed, and was informed by, our ongoing systematic review [16]. For example, the addition of domain 1 to the NASSS framework was prompted by a strong theme in our empirical data that non-adoption and abandonment of technologies were often explained by heterogeneity and unpredictability in the patient’s illness, co-morbidities and socio-cultural background. The addition of Case F (data warehouse) was prompted by the discovery from our secondary research that technologies intended for sharing data between organisations raised unique logistical, technical and professional challenges.

For each of the six case studies, we analysed qualitative data thematically and produced an initial narrative summary. We wove in quantitative data (e.g. uptake and usage rates) as part of that narrative and used longitudinal methods (e.g. repeat interviews, data trends) to build the narrative over time. We held a series of meetings (approximately monthly) to discuss each of the NASSS domains, singly and in combination, as they pertained to each case study—and tested emerging theory against these domains. Our refinement of the NASSS framework, and in particular the generation of key sub-questions within each domain, owed much to these cross-case meetings.

## Results

Below, we apply the different domains of the NASSS framework to our case studies before considering (in the Discussion) the implications of our findings in terms of complexity theory. We have presented Case A (video outpatient consultations) in depth and added additional data from other case studies where they add to the granularity of the analysis.

### Domain 1: the condition

Differences in the underlying illness largely explained differences in the fortunes of the video consultation option in the four services studied in Case A. Routine check-ups for adults with diabetes and follow-up consultations after cancer surgery were mostly consistent and predictable (i.e. simple), and most unpredictable eventualities were of low risk. By the end of the study period, approximately 20% of all consultations in these clinics were being conducted by video.

In contrast, diabetes in pregnancy was an example of a complex condition. In pregnancy, diabetes tends to be metabolically volatile and if poorly controlled may lead to foetal abnormalities or death. Many pregnant women had developed diabetes only since becoming pregnant, and so were novices in self-administering insulin. The lead physician felt strongly accountable to the unborn child, and so only offered the video option to patients (3% of the total) judged to be ‘low risk’ (for example, those with high health literacy, good technological skills and fluency in English).

Heart failure is a serious, unpredictable and often unstable (hence, complex) illness whose effects vary from patient to patient and in the same patient over time [46]. It is mostly a condition of older people and occurs disproportionately in lower socio-economic groups. One of its common side effects is profound tiredness, and it is almost always associated with other co-morbidities (notably kidney disease, diabetes, depression or cognitive impairment). Heart failure nurses in our study made judgements about the stability or otherwise of the illness and about patients’ co-morbidities (including cognitive ability and mental health status), health and technological literacy, family support and technical set-up at home and motivation. As a result, the video consultation option was deemed inappropriate for many (at the time of writing, fewer than 20 such consultations had been undertaken across a clinic population of several hundred).

Complexity in the underlying condition was also associated with non-adoption, abandonment or limited usefulness of technologies in Cases B and C (in which dementia or multi-morbidity respectively made the patient unable or unwilling to use the supplied technology) and Case F (in which the predictive risk modelling tool selected multi-morbidity as a risk factor for hospital admission, but few such patients proved to be ‘textbook cases’ to manage).

### Domain 2: the technology

The technologies used for video consultations, Skype™ and FaceTime™, are both mass-market software packages from large multi-national companies, presenting low risk of supplier withdrawal and relatively straightforward substitutability (hence, in these respects they could be classified as simple or complicated). However, there were elements of complexity. For example, they were run from NHS hardware (sometimes many years old) and from patient-held laptops, tablets or smartphones of varying quality and dependability. The software was sometimes logistically difficult to install on NHS computers (e.g. because of limited capacity of the IT support team and maintain ‘non-standard’ software environments), and even when installed, it was not 100% dependable for both technical (machine ‘crashing’) and human error (e.g. forgotten password) reasons. Workarounds tended to use low-tech, dependable solutions (e.g. community heart failure nurses defaulted to telephone consultations when video connection failed), thus reducing complexity.

Whilst video transmits speech and visual cues reasonably well (although variations in audio and video quality are quite common), clinical examination sometimes needs other modalities. In heart failure care, for example, the physical examination (e.g. blood pressure, heart rhythm, leg oedema) that the nurses considered essential was not easy in the remote environment—though it was sometimes possible with patient and carer assistance when the nurse knew the patient well. There was no easy or automated way of sharing and recording patient-held data such as home blood pressure readings (patients typically read out numbers but some misinterpreted the digital display or viewed it upside down).

The other case studies illustrated different aspects of complexity in the technology. GPS tracking technology (Case B), pendant alarms (Case C) and care organising software (Case E) all relied on bespoke solutions from small and medium-sized enterprise (SME) companies, and hence were vulnerable to withdrawal; these companies sometimes lacked capacity to meet the bureaucratic requirements for potential spread and scale-up. Case D used software that had been developed as part of an academic research study; importantly, the research nurse and technical team were co-located, allowing minor (but potentially critical) technical issues to be resolved in an ongoing way. Case F featured bespoke software supplied through a longstanding relationship with an SME that was subsequently acquired by a global company. These technologies all required considerable knowledge and skill to use to their full potential. An assumption underpinning the design of patient-held assistive technologies was that a group of relatives and/or friends would exist, live locally, be technology-savvy and be able and willing to collaborate around the care of the index case. In fact, such networks were rarely pre-existing; they often had to be built and nurtured. Cases B, C and D highlighted the role of both lay carers and professional staff in helping to set up and ‘service’ the technology and keep it in working order, a role that could be particularly onerous if the technology (as in case B) was not dependable.

The data warehouse for integrated case management in Case F was well embedded organisationally (in the sense that it was enshrined in national policy and local sub-contracts and data sharing agreements, and staff were employed to work on it). But it was not *technically* well embedded in the sense of seamless interoperability of data between participating organisations; significant workarounds were required to (for example) share care plans. The predictive risk modelling tool generated a different kind of risk estimate than a home visit from a clinician or social worker who knew the individual well and who had the capacity and authority to bear witness to a narrative and make contextual judgements. Our data illustrated that often neither approach alone offered the full picture that was sometimes necessary for making judgements. Thus, the output (risk score) generated by the technology was complex (in the sense that it was incomplete and contested).

### Domain 3: the value proposition and overall value chain

The supply-side value proposition for video consulting in Case A currently appears complex. The multi-national companies behind Skype™ and FaceTime™ are also developing multiple other health products, especially directed towards the expanding ‘wellness and wearables’ market. From a purely financial perspective, such direct-to-consumer products may offer a more lucrative supply-side value proposition than investing in a major business venture to support video consultation products and services in the NHS, because working through technical and information governance challenges is a resource-intensive and time-consuming process with no guarantee of meeting shareholder or executive expectations at the end of it. Our interviews also suggest that companies are aware of the potential for reputational risk associated with seeking to profit from virtual consultations in the NHS.

The demand-side value (to patients) of video consultations is also complex, since the evidence base on which it rests is currently sparse. Whilst around 20 randomised controlled trials in a range of conditions have demonstrated equivalent efficacy and safety between video and face-to-face consultations [38], the samples for these trials are likely to have been carefully selected. Members of our VOCAL patient advisory group raised concerns about the risk of a ‘two-tier’ service in which demand-side value for a minority of patients will be gained at the expense of service cuts for the majority—a concern which, though speculative, has recently been echoed by professional bodies, clinicians and patients [47, 48].

In Case D, one aspect of the value proposition (which affects both supply- and demand-side value) is the potential of the data collected to inform the development of predictive algorithms based on biomarker changes over time and hence predict and pre-empt decompensation, hence averting hospital admission (rather than just prompting medication changes on the basis of, say, a rise or fall in blood pressure). This option creates new possibilities for ‘personalised’ medicine, but it also increases complexity. Whilst real-world value is hard to assess in the context of a randomised controlled trial, we note that the promise (or aspiration) of the telehealth package in Case D is highly complex, since it seeks to achieve multiple goals, including: (1) improving heart failure management in the community; (2) reducing demand on services (by allowing nurses to take on higher case loads); (3) preventing unplanned admissions; and (4) developing further predictive capabilities.

Case E illustrates two very different business models (and technology development models) for similar technologies and use cases. In one (Product A), the value proposition was highly speculative and little attempt was made to work with intended end-users to increase the technology’s fitness for purpose (and hence its desirability) during development. The implicit assumption was that the technology would be more or less plug-and-play; the business model rested on provider organisations paying for a block contract even though the intended benefits (and/or savings elsewhere in the system) were not clear. Product B included substantial up-front investment (from publicly funded R&D) to undertake co-design work; it was explicitly developed by a charity as a ‘public good’ in which costs to end-users would be minimal and viewed as a *component* of a wider (and ongoing) charity-supported package. These projects are both ongoing, but at the time of writing we would classify Product A’s value proposition as complex and Product B’s as complicated.

### Domain 4: the adopter system

In the adoption of video consulting (Case A), there was considerable complexity in the adopter system. There was, for example, a striking difference between innovators (who embraced the new technology and way of working with enthusiasm) and other clinicians on the same teams who were reluctant to change, reflecting previous research showing that staff resistance is the single most important reason given for low uptake of remote health care [10, 49]. The hurdle was not merely learning to use a new technology but also accepting changes in identity (e.g. some staff did not view themselves as ‘techy’) and role (e.g. helping a remote patient troubleshoot technical problems with Skype™ or FaceTime™), dealing with perceptions of overload when running a new virtual service in parallel with a traditional face-to-face one and feeling under pressure to realise efficiency gains before the system had been fully redesigned to maximise such gains. New staff roles were not restricted to clinicians directly using the video technology; receptionists, clerks and technicians all had to accommodate new roles that the technology required in order to ‘work’. The same was true of the patient, who could sometimes but not always seek technical help from family members.

The other case studies illustrated additional complexities in the adopter system, such as some staff expressing ethical reservations about ‘tagging’ clients (Case B) or clinical concerns about the data generated by the technology (Case D—some clinicians were worried about possible legal liability if telehealth data, generated elsewhere and impossible to verify directly, were later found to be flawed). In Case C, clients sometimes rejected a pendant alarm because it symbolised dependence or because they were unwilling to pay a small monthly connection fee or be placed in a dependency relationship with a relative or neighbour.

### Domain 5: the organisation(s)

The hospital trust that hosted the VOCAL study had strong leadership and good managerial relations; it met key criteria for technological innovativeness (e.g. it had previously won a national ‘Digital Trust of the Year’ award), and there was board-level enthusiasm for the introduction of video consultations. Despite these encouraging preconditions (‘simple’ in our taxonomy), other features of the organisation were highly complex. In particular, it had very limited spare staff time and resources (e.g. key posts were unfilled, and many clinic terminals were running outdated versions of software packages)—a problem known as ‘lack of organisational slack’ [18]. In addition, whilst many senior decision-makers *assumed* that the new service would save money by making services more efficient, the question of whether a video consultation would *actually* cost less to deliver was not easy to answer because of potential knock-ons in the system (e.g. the need for additional IT support and staff training; the fact that rooms still needed to be occupied, records retrieved and appointments booked even when the consultation was virtual; and the theoretical possibility of an increase in appointments as clinicians and patients found it easier to connect).

Another feature of complexity was that whilst video consultations between clinicians and selected patients usually worked well, the linked routines for booking appointments, managing the clinic list (e.g. registering when each patient had ‘arrived’ and ‘left’) and organising follow-up did not mesh well with a system that had evolved to process patients using their physical presence (waiting in line at a reception desk), manual transfer of paper records between different plastic ‘bins’ and sticky notes. Alignment with such routines was initially achieved using workarounds; by the end of the study, new (computer-based) routines had been developed by some but not all participating teams. Whilst Skype functionality increased access (by, for example, allowing patients to send messages to the clinician’s Skype account), and whilst this ease of access was sometimes clinically appropriate and encouraged (e.g. “drop me a message to confirm the change of insulin dose was OK”), it generated complexities elsewhere in the system, since the clinician then had to log the message on the medical record.

Similar complexity-related challenges were evident in the community heart failure study. The community trust was a digital innovator and had supplied all nurses with tablet computers to help them with various aspects of their work. Again, whilst video consultations worked well *clinically* for selected patients, at the time of writing they were not well integrated *logistically* with the administrative aspects of the service. There was no formal opposition from top management in the community trust to the nurse-led video consultation service. But neither was there strong enthusiasm, and there was limited spare capacity among front-line teams to undertake the work of making sense of the new approach, enrolling and training staff beyond initial enthusiasts, implementing new work routines and evaluating the service.

In our other case studies, enthusiasm at board level was sometimes absent (e.g. because business cases were weak) and/or key opponents were strategically placed and had high wrecking power (specific examples withheld). In Case D, wide variability in clinician engagement among the different study sites could be explained largely in terms of the extent to which the local team shared a vision for how remote biomarker monitoring for heart failure might enhance rather than threaten the existing service, an aspect of implementation work that May calls ‘coherence work’ [22].

In Case F, establishing integrated case management through shared data and predictive risk modelling technology was extremely complex because multiple organisations needed to be involved; the establishment and development of the programme unfolded over several years and relied on partnership working and contracting arrangements at different levels of multiple organisations. The technology was implemented, but the anticipated reduction in costs from reducing hospital admissions were not realised as real savings because of the complexities of reimbursement mechanisms and because case management was not always successful in avoiding admissions.

### Domain 6: the wider system

The most significant system-level challenge to the scale-up and spread of video consultations in Case A was that there was no established national tariff for funding such consultations. In the VOCAL study, the local commissioning organisation proposed reimbursing such consultations at a rate (‘pass through tariff’ [50]) intermediate between a telephone consultation and a face-to-face one. But even though members of the relevant national policymaking team were on the study steering group and there was no opposition ’in principle’ to establishing a national tariff, this had still not been achieved at the time of writing—mainly because data to inform costing calculations were difficult to obtain and contested by some parties. This is a good example of how innovative, technology-supported service models can succeed as demonstration projects through local workarounds but will fail to spread or be sustained unless the regulatory and financial context is supportive [51]. Another contextual factor which added complexity in case study A was a mixed reaction from professional bodies, whose enthusiasm for new, potentially more efficient, models of care was tempered by concerns about workload and threats to equity.

Our national-level stakeholder interviews revealed another aspect of complexity relating to the nature and strength of evidence expected by different stakeholder groups. The technology industry typically moves quickly, with a development model based on rapid iterations of technologies and a pragmatic understanding of what works in practice (the ‘fail early, fail often’ principle). Policymakers, in contrast, tend to want what they call ‘gold standard’ evidence (for example, from randomised controlled trials) to ‘prove’ that a particular technology has the impacts claimed. This mismatch appeared to explain some of the slow progress on national-level policy in relation to video consultations.

The acute trust where our video consultation study was based was one of the first public sector providers in the UK to introduce this service model [39]. In the last year of the VOCAL study, more than 50 organisations contacted the lead clinician seeking advice or asking to visit to see the video consultation service in action. This is an example of the important role of inter-organisational networking in supporting the exchange of both explicit and tacit knowledge [18].

### Domain 7: adaptation over time

The video consulting services in Case A illustrated both resistance to adaptation (through material limitations and institutionalised information governance regulations) and adaptiveness (through clinicians’ creative and responsive use of the technology). The NHS has a ‘locked-down’ computer environment in which any new hardware or software must be carefully considered and formally approved before being installed or upgraded (a characteristic that reduces complexity for IT managers but tends to increase complexity for front-line staff). Rapidly evolving software sits awkwardly in such an environment. In the VOCAL study, an automated upgrade to Skype™ made the system non-functional on clinical terminals until re-authorised by someone with administrator-level access rights—a problem that resulted in some remote clinics having to be done by telephone.

The material features of Skype™ enabled the development of ‘ad hoc’ consulting in the young adult diabetes clinic, for example, when the patient saw that the clinician was online and sent a text (SMS) message asking a question about a recently changed insulin dosage. The clinician could either reply by SMS message (within Skype™) or offer a real-time video consultation (typically very short). This adaptive use of video technology for patient-initiated consultations was viewed by clinicians as a game-changer for ‘challenging’ patients (characterised by high non-attendance rate at clinic, poor glycaemic control and a history of hospital admission for diabetic emergencies).

Several other cases in our dataset illustrated a similar tension between system rigidity (for contractual or cost reasons) and adaptation (through user creativity). In Case C, for example, potentially remediable problems occurred with some alarms, but adaptation was impossible because of the risk of loss of warranty. A pendant alarm service initially introduced to provide *emergency physical support* (e.g. for falls) was adapted over time to provide *non-emergency emotional support* for certain older people, who were encouraged by call centre staff to press the alarm button to trigger a supportive conversation when feeling lonely.

In Case F, following the introduction of the integrated case management data warehouse technology, clinical and administrative staff across different organisations collectively learnt and redefined what this technology could and could not do. They amended, adapted and worked around it. For example, clinicians and practitioners reviewed the outputs of the data-driven risk stratification model but also supplemented them with other data and used their judgement to target additional patients who had *not* been flagged as ‘high risk’ by the technological algorithm. Technical developers continually updated the user interface through an ongoing relationship with the procuring body. Notwithstanding these efforts, there was a brittleness about the technology (and the work routines it presupposed) that staff experienced as persistently frustrating. Thus, there was a sense that the technology had been ’successfully’ implemented and was for the moment being sustained (because it met policy expectations), but was not truly fit for purpose.

## Discussion

Through in-depth, longitudinal ethnography across a maximum-variety sample of local technology-supported innovations, along with an analysis of national context, we have shown that failures, partial successes and unanticipated problems are common. Using the NASSS framework, these outcomes can be explained by complexity across multiple, interacting domains. Technology-supported innovation programmes face particular challenges when:

- *The condition or illness* is complex because it is poorly characterised, poorly understood, unpredictable in its natural history or associated with multiple co-morbidities or socio-cultural concerns (such as poverty, low health literacy or particular beliefs or traditions).
- *The technology* creates additional complexity because it has multiple interacting components, requires close embedding within already-complex technical systems, lacks dependability, provides an unreliable, incomplete or contested picture of the condition, requires advanced knowledge to use it or exists only as a bespoke solution that is vulnerable to supplier withdrawal.
- *The supply-side value proposition* rests on an underdeveloped, implausible or risky business case (hence, is unlikely to attract investment), or the *demand-side value proposition* suggests that (from the patient’s perspective) the technology could be undesirable, unsafe, ineffective or unaffordable.
- *The adopter system* is complex because the innovation does not merely require staff to take on new roles but also puts staff under pressure, threatens their professional identity, values or scope of practice, or poses a risk of job loss; because it requires patients to undertake complex tasks such as initiate changes in therapy or make judgements about what is an emergency; or because it presupposes a network of carers who are willing and able to coordinate their input.
- *The organisation(s) is/are complex* as a result of severe resource pressures (e.g. frozen posts), weak leadership and managerial relations and a climate in which creativity and risk-taking are punished; and in situations where, in relation to this particular technology, there is minimal tension for change, poor innovation-system fit and multiple opponents to the programme, some of whom are strategically placed and have wrecking power. Complexity will loom large when new team routines or care pathways predicated on the new technology conflict with established ones, and when significant work is needed to build shared vision, engage staff, enact new practices, monitor impact and support ongoing adaptation. It will be a prominent feature of a programme spanning multiple organisations who have no formal links and/or have conflicting agendas, where funding depends on cost savings across the system, where the costs and benefits to each partner organisation are unclear, and where new infrastructure for the proposed programme conflicts with existing infrastructure and where there are significant budget implications.
- *The wider system* is complex because policy changes that the new service model requires raise tricky political, regulatory, legal, financial or other challenges, because policymakers and industry have different views on what counts as high-quality evidence, because professional bodies and lay stakeholders are currently unsupportive or opposed or because there are barriers to inter-organisational networking and knowledge-sharing.
- *The time dimension* is complex because further adaptation and/or co-evolution of the technology or service is impossible (or only possibly to a limited extent), or because sense-making, collective reflection and adaptive action are discouraged in a rigid, inflexible implementation model.

As the case narratives above illustrate, when there is complexity across *multiple* domains (and this occurs commonly), outcomes become even less predictable, less controllable and (hence) less amenable to conventional planning and implementation logic.

Despite abundant evidence of complexity in multiple domains in all our case studies, our data indicated a tendency of planners, policymakers and technology designers to assume that the issues to be addressed were merely *complicated* (hence, knowable, predictable and controllable) rather than *complex* (that is, inherently not knowable or predictable but dynamic and emergent). What might be called ‘complexity work’ (adaptation and adjustment to accommodate a host of emergent issues) was absent from policymakers’ version of the project but loomed large in the day-to-day experience of front-line staff.

A complicated programme can be managed rationally by careful planning, implementation of agreed procedures and monitoring. Typically, such programmes are divided into discrete work packages (perhaps ’work-as-imagined’ packages [52]), each of which can be defined, assigned a leader, undertaken and reported on separately from other work packages. A focus on complicatedness rather than complexity is illustrated, for example, by the digital maturity assessment tools produced by NHS England [53]. The ’de facto standard in UK government’ for managing complicated programmes, underpinned by a logic model, is PRINCE2 (PRojects IN Controlled Environments) [54]. PRINCE2 and similar tools focus almost exclusively on an abstracted depiction of process (what needs to be done, by whom and by when).

A *complex* programme, especially one that is designed around clinical or social care of sick or vulnerable people, requires a very different approach. Its management must attend carefully to people, motivations, values and professional norms, and put mechanisms in place to detect deviations from expected outcomes, identify the numerous contributory causes and make timely adjustments by adapting technologies, practices and workflows. This is partly about a different, more flexible, iterative and user-centred approach to programme management (which some have termed ‘co-realisation’ [55]), partly about a central focus on the *people* involved, including the deeply held professional identities, norms and values that underpin so-called resistance to new technologies [21] and the need for organisational members to *make sense* of technology-supported change in an ongoing, evolving way [23], and partly about the importance of *organisaton and system learning* to ensure that what has been learnt during deployment can be captured and re-used to inform strategies for subsequent scaling up and sustainability [56].

The uncertainty of outcomes in complex programmes means that they are highly likely to witness active experimentation by users as they grapple with the challenges of discovering the capabilities and limitations of a new technology. These experiments might involve reconfiguring the technologies, processes or both. Hence, the phase within a programme that is commonly referred to as ‘deployment’ needs to be viewed not as its final denouement but as an opportunity for learning, refinement and adaptation. The key to achieving this is the use of a variety of modes of communication, both among programme team members and between the programme team and the users [57].

It is encouraging that some recognition of the need for technology ‘deployment’ to be iterative, adaptive, people-focused and oriented to social learning is evident in some recent initiatives in healthcare. For example, the NHS Technology Adoption Centre (NTAC), which is part of the UK’s National Institute for Health and Care Excellence (NICE), provides innovation process guides that are designed to capture and distill the experiences of early adopters [58]. However, NTAC’s primary focus is on tackling organisational issues such as stakeholder recruitment and business case development, rather than, for example, supporting the day-to-day work of monitoring, communicating, evaluating and adapting.

## Conclusion

This first empirical application of the NASSS framework has illustrated how complexity characterises multiple dimensions of many technology-supported change programmes. In every case study, the mismatch between work-as-imagined and work-as-done was substantial. Illnesses behaved idiosyncratically, not as depicted in textbooks. Technologies showed promise (including potential value for developers and patients) but also both symbolic and material fickleness. Human agents (staff, patients, technology developers, policymakers) brought their values, motives, capabilities and beliefs to bear on their assessment of local situations, and this affected their resulting action (or inaction), which then had knock-ons across the system. The organisational and wider setting for introducing, implementing and monitoring technologies was characterised by both opportunities and constraints that were multiple and changing. Creative, adaptive solutions and workarounds sometimes but not always helped keep the show on the road.

We conclude that a rationalist approach to implementing technology programmes, based on abstracted principles for managing complicatedness in linear and static deployment models, is unlikely to lead to the large-scale ‘disruptive innovation’ that policymakers have envisaged, nor will it address the specific challenges of local scale-up, distant spread and long-term sustainability. As Ludwig Wittgenstein commented in ‘Philosophical Investigations’,

*We have got onto slippery ice where there is no friction and so in a certain sense the conditions are ideal, but also, just because of that, we are unable to walk. We want to walk, so we need friction. Back to the rough ground!* [59].

Our data suggest that it is towards this ‘rough ground’ of real-world implementation, collective sensemaking and social learning at the front line that attention should now be turned. Until recently, researchers trod tentatively if at all on such ground, but the shift to complexity thinking in the business and management literature has begun to generate principles, tools and practical approaches that are (at least to some extent) evidence-based.

Whilst we originally developed the NASSS framework to support academic activity (e.g. ‘conjunctive theorising’ [35] to illuminate and explain our case studies), we have begun to use the framework in more practical ways to try to increase the success of complex programmes in health and social care. Relevant to this practical application (which is currently in its early stages) is the work of Janssen et al. on technology-driven transformation [60]. These authors acknowledge that complexity theory eschews universal solutions and predictive models but maintain that there are nevertheless some core principles of non-linear system change that will increase the chances of programme success.

Adapting these suggestions and taking account of the findings presented here, we propose the following principles for technology adopters, commissioners and policymakers: (1) assess the nature and extent of complexity in the programme and ensure that emergent and adaptive measures are used to address these issues; (2) establish overall leadership (since complex programmes often suffer from outsourcing of control and coordination); (3) craft and sustain a vision (ensure that key players understand and share a sense of why the project is important); (4) create incentives (but leave front-line staff to work out how to deliver); (5) respond adaptively as the programme-in-context evolves (for example, by collecting and reflecting on emerging data and harnessing human creativity); (6) control growth (since projects that evolve organically are vulnerable to over-ambitious extension and scope creep); (7) create slack (to resource adaptive responses); and (8) manage the tension between innovation and implementation, especially when continuing evolution of the technology (e.g. additional functionality) adds to complexity.

Our empirical findings also suggest that it will often be mission-critical to reduce complexity in as many domains and sub-domains as possible. Maylor et al., focusing mainly on commercial projects, recently developed a complexity assessment tool intended to be used prospectively to identify, understand, reduce and/or ‘run with’ the different aspects of complexity in a technology project or programme [36]. We are currently in the process of using the NASSS framework to adapt this tool to support a systematic approach to complexity reduction in the very different context of health and social care.
